# Emerging roles of lipocalin-2–mediated neuroimmune interactions in chronic pain and itch

**DOI:** 10.3389/fphar.2026.1799602

**Published:** 2026-04-20

**Authors:** Shiyu Sun, Jiahui Lin, Xuelong Wang, Tong Liu, Guokun Zhou, Peipei Kang

**Affiliations:** 1 Institute of Pain Medicine and Special Environmental Medicine, Nantong University, Nantong, Jiangsu, China; 2 Department of Thoracic Surgery, Capital Medical University Electric Power Teaching Hospital, Beijing, China; 3 Department of Anesthesiology, Affiliated Tumor Hospital of Nantong University & Nantong Tumor Hospital, Nantong, Jiangsu, China

**Keywords:** glial cells, itch, lipocalin 2, neuroimmune, pain

## Abstract

Neuroimmune interactions serve as a core regulatory node of chronic pain and pruritus and a key target for clinical intervention. Lipocalin-2 (LCN2), a member of the lipocalin superfamily, is a multifunctional protein widely expressed in various tissues and cells. LCN2 exerts diverse biological effects by regulating iron metabolism, mediating inflammatory responses, and participating in signal transduction pathways. In recent years, accumulating evidence has indicated that LCN2 plays a crucial role in the pathogenesis of chronic pain and pruritus through neuroinflammation, neuron-glia interactions, and modulation of neural signaling. In chronic pain, LCN2 contributes to the development and maintenance of inflammatory pain, neuropathic pain, morphine tolerance, and thalamic pain by disrupting iron homeostasis, inducing oxidative stress, and promoting central sensitization. For chronic pruritus, LCN2 modulates the excitability of pruritus-related neurons via pathways such as the IL-6/STAT3 axis, and participates in the pathological processes of pruritus in allergic contact dermatitis, xerosis, atopic dermatitis, and psoriasis. This review summarizes the structural characteristics, physiological functions of LCN2, and its specific mechanisms in regulating chronic pain and pruritus, and further discusses the potential therapeutic value of targeting LCN2, aiming to provide a theoretical basis for the development of novel interventions for chronic pain and pruritus.

## 1 Introduction

Lipocalin-2 (LCN2), or neutrophil gelatinase-associated lipocalin (NGAL), was first isolated from neutrophil granules at sites of human inflammation and from mouse renal cells. Beyond its primary source in activated neutrophils, LCN2 is now known to be expressed in diverse tissues and cells, including the liver, adipose tissue, and the central nervous system. It performs varied functions, contributing to processes such as iron metabolism, inflammatory responses, and tumorigenesis (Živalj et al., 2023).

While classical neuroimmune mediators such as IL-1β, TNF-α, and CCL2 function as direct signaling ligands that bind transmembrane receptors to trigger rapid intracellular cascades, LCN2 belongs to the lipocalin superfamily and operates through distinct biochemical mechanisms. Unlike these small cytokines, LCN2 possesses a unique β-barrel structure with a central hydrophobic cavity that sequesters iron-bound siderophores to modulate systemic and local iron homeostasis (Schröder et al., 2023). Furthermore, whereas CCL2 primarily acts as a chemoattractant for immune cell recruitment, LCN2 functions as a versatile microenvironmental fine-tuner; it stabilizes matrix metalloproteinase-9 (MMP9) to prolong extracellular matrix degradation (Cavalcanti et al., 2025) and acts as a metabolic stressor that sensitizes neurons via receptors such as 24p3R and MC4R (Mosialou et al., 2017; Dekens et al., 2021). Positioning LCN2 as a central orchestrator rather than a transient inflammatory marker is therefore essential for understanding its sustained role in the transition from acute physiological defense to chronic pathological sensitization.

The transition from acute sensation to chronic pathology originates with early pathogenic events, defined by the activation of peripheral nociceptors and pruriceptors following tissue injury or allergen exposure. In the acute phase, noxious stimuli open ion channels such as TRPV1 and TRPA1 and prompt the release of neuropeptides like substance P and calcitonin gene-related peptide (CGRP), initiating local neurogenic inflammation (Bautista et al., 2013; Gouin et al., 2017). However, persistent or repetitive stimulation ultimately precipitates a fundamental shift in the functional state of the nervous system. This process of transition from an acute to a chronic state involves an evolution from purely peripheral-driven mechanisms to central sensitization. During this transition, the spinal cord and supraspinal centers exhibit heightened reactivity, even in the absence of sustained peripheral input. While the initial inflammatory response is primarily driven by the transient release of cytokines, the subsequent chronic pathological state may sustained through LCN2-mediated mechanisms—including the dysregulation of iron homeostasis and the remodeling of dendritic spine morphology (Mucha et al., 2011). In the later stages of the disease, LCN2 is secreted by spinal astrocytes, thereby establishing a state of centrally autonomous sensitization.

Pain and itch represent distinct, complex sensory experiences. While both involve neural network activation, their physiological mechanisms differ: pain typically acts as a protective response to harmful stimuli, whereas itch is primarily an unpleasant sensation. Recent research has increasingly focused on the role of LCN2 in nervous system function, particularly its potential mechanisms in modulating pain and itch. Evidence indicates that LCN2 may regulate sensory functions through neuroinflammation, glial cell interactions, and the modulation of neural signaling pathways. This article reviews current advances in understanding LCN2 in chronic pain and itch and considers its potential therapeutic relevance, providing a foundation for further mechanistic studies and novel interventions.

## 2 Structure and receptors of LCN2

LCN2 is an extracellular small-molecule protein with a relative molecular mass of 25 kDa. The human *LCN2* gene, located on chromosome 9q34.11, contains seven exons and encodes a 198-amino acid protein, which includes a signal peptide, the mature protein comprises 178 residues. Similarly, the mouse *Lcn2* gene is situated on chromosome 2p25 and encodes a 200-amino acid precursor, with the mature protein containing 180 residues. As a member of the lipocalin family, LCN2 features a characteristic β-barrel domain that binds small-molecule ligands such as iron-bound bacterial siderophores, playing an important role in innate immunity and iron metabolism (Bachman et al., 2009). The domain architecture of LCN2 is highly conserved across humans, mice, and rats. Its N-terminal signal peptide, approximately 20 residues in length, is essential for secretion and is adjacent to the canonical lipocalin ligand-binding domain, together forming the protein’s core functional region (Bachman et al., 2009). The three-dimensional structure of LCN2 is also highly conserved, with its core formed by eight anti-parallel β-strands that create a barrel-like scaffold enclosing a cup-shaped ligand-binding cavity. This architecture provides precise molecular recognition, allowing LCN2 to bind diverse specific ligands through stereochemical complementarity and thereby execute characteristic lipocalin functions (Hu et al., 2018; Schröder et al., 2023).

Compared to other lipocalins, the ligand-binding pocket of LCN2 exhibits a larger volume and higher surface polarity. This distinctive structure not only promotes efficient binding to plasma membrane receptors but also enables the specific recruitment of larger, less hydrophobic protein ligands via multivalent interactions, leading to the assembly of multi-component complexes. The dynamic nature of these complexes allows LCN2 to significantly influence cell cycle control networks and signal transduction pathways. Furthermore, LCN2 has been shown to bind matrix metallopeptidase 9 (MMP9), low-density lipoprotein receptor-related protein 2 (LRP2), Toll-like receptor 4 (TLR4), and 24p3R, thereby mediating various inflammatory and immune processes (Qian et al., 2005; Schröder et al., 2023). These unique structural characteristics equip LCN2 to perform diverse biological functions both intracellularly and extracellularly, while also acting as a signaling molecule that fine-tunes immune and inflammatory responses.

To date, at least six putative LCN2 receptors have been identified: NGALR (SLC22A17/24p3R), LRP2 (megalin), LRP6, and melanocortin receptors (MC4R, MC1R, MC3R) (Schröder et al., 2023). NGALR, a transmembrane transporter, binds apo-LCN2 with high affinity, mediating iron uptake/efflux and apoptosis, though binding affinity data vary by experimental method (Devireddy et al., 2001; Cabedo Martinez et al., 2016; Thévenod et al., 2023). LRP2, a large multi-ligand endocytic receptor, interacts with LCN2 via electrostatic forces, regulating its renal excretion and participating in nutrient transport (Barasch et al., 2016; Christ et al., 2016). LRP6 binds mouse LCN2 to inhibit Wnt/β-catenin signaling, but cross-species validation is lacking (Jiang et al., 2023). Among melanocortin receptors, MC4R has the highest LCN2 affinity, mediating appetite and energy metabolism regulation by activating cAMP signaling (Tao, 2010; Mosialou et al., 2017). In astrocytes, when LCN2 binds its receptor, a preferential activation of the NF-κB pathway over the JAK/STAT3 pathway is generally considered to promote differentiation toward the A1 reactive subtype (Lee S. et al., 2011). Given that LCN2 expression is elevated in A1 astrocytes but relatively lower in the A2 subtype, this differential abundance suggests a close relationship between LCN2 levels and the signaling pathways it drives or modulates (Jung and Ryu, 2023). We therefore hypothesize that the selective activation of the NF-κB versus JAK/STAT3 pathways in astrocytes may be regulated by, or dependent on, LCN2 concentration (Table 1).

**TABLE 1:** LCN2 receptors, cellular distribution, and functional outcomes.

Receptor	Cellular distribution	Functional outcomes
NGALR (SLC22A17/24p3R) (Devireddy et al., 2001; Cabedo Martinez et al., 2016; Thévenod et al., 2023)	Spinal astrocytes, neurons	Mediates intracellular iron transport; activates the NF-κB signaling axis to induce pro-inflammatory cytokine release (e.g., IL-1β, TNF-α).
LRP2 (Barasch et al., 2016; Christ et al., 2016)	Renal tubular epithelial cells, choroid plexus	Regulates LCN2 reabsorption and renal excretion; participates in endocytosis of various ligands and nutrient transport.
LRP6 (Jiang et al., 2023)	Mesenchymal cells, embryonic fibroblasts	Interacts with LCN2 to inhibit the Wnt/β-catenin signaling pathway, thereby modulating osteogenic differentiation.
MC4R (Tao, 2010; Lee S. et al., 2011; Mosialou et al., 2017; Jung and Ryu, 2023)	Hypothalamic neurons, DRG neurons, spinal neurons	Regulates appetite and energy balance; enhances neuronal excitability by sensitizing TRPV1 channels or antagonizing the inhibitory effect of morphine on calcium currents.

## 3 Physiological functions of LCN2 and associated diseases

**FIGURE 1:**
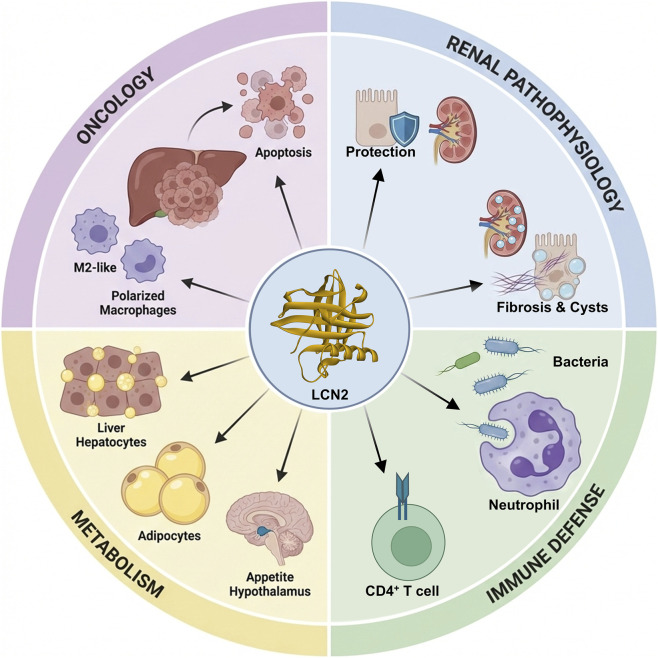
Regulatory mechanisms of LCN2 in different diseases. During the progression of chronic kidney disease, LCN2 is continuously induced via the EGFR-HIF-1α pathway. It acts as a mitogenic factor that promotes the abnormal proliferation of renal tubular epithelial cells, driving cyst formation and the advancement of renal interstitial fibrosis. In hepatoma cells, LCN2 activates the mitochondrial apoptotic pathway and induces a caspase cascade. Within the tumor microenvironment, LCN2 reshapes macrophage polarization to promote a shift toward the M2 phenotype, thereby fostering an immune milieu conducive to tumor growth. Osteoblast-derived LCN2 binds to the melanocortin-4 receptor (MC4R) in neurons of the hypothalamic paraventricular and ventromedial nuclei, which subsequently activates the MC4R-dependent anorexigenic pathway. LCN2 also induces immune tolerance by upregulating the expression of the immune tolerance molecule human leukocyte antigen-G (HLA-G) on CD4^+^ T cells from healthy individuals and by promoting the expansion of regulatory T cells (Tregs).

LCN2 is a multifunctional acute-phase protein with complex biological roles in systemic homeostasis and diverse pathological processes (Figure 1). In inflammatory networks, pro-inflammatory stimuli like lipopolysaccharide (LPS) and tumor necrosis factor-α (TNF-α) potently induce LCN2 expression. This upregulation amplifies inflammation and enhances the chemotactic and activation capacities of neutrophils and macrophages through positive feedback, thereby driving inflammatory progression (Wan et al., 2022). LCN2 further promotes the secretion of cytokines such as IL-1β and IL-6 from inflammatory cells via activation of the MAPK/NF-κB signaling axis, creating an inflammatory amplification loop. The specific binding of LCN2 to MMP9 is key to atherosclerotic plaque instability. This complex degrades the extracellular matrix and weakens the fibrous cap, significantly increasing plaque rupture risk and representing a likely mechanism for LCN2-mediated pathology in cardiovascular disease (Hemdahl et al., 2006).

In renal pathophysiology, LCN2 is a well-established early and sensitive biomarker for acute kidney injury (AKI) (Bennett et al., 2008). Its urinary concentration rises 24–48 h before serum creatinine. During early AKI, LCN2 activates autophagy via crosstalk between HIF-1α and NF-κB signaling, protecting renal tubular epithelial cells from apoptosis and exerting a renoprotective effect (Qiu et al., 2018). In contrast, during chronic kidney disease (CKD) progression, sustained LCN2 expression is induced via the EGFR-HIF-1α pathway, where it acts as a mitogenic factor to promote abnormal tubular epithelial cell proliferation, driving cyst formation and renal interstitial fibrosis (Viau et al., 2010). This duality reflects LCN2’s function as a stress-response protein: it provides protective compensation acutely, while chronic activation transforms it into a pathological pro-fibrotic factor, creating a functional switch from protection to injury. This shift provides a rationale for time-window-specific therapeutic strategies targeting LCN2.

As a key innate immune effector, LCN2 enhances phagocyte recognition and clearance of Gram-negative bacteria via a unique LPS-binding site. Crystallographic studies show that LCN2’s hydrophobic binding pocket specifically captures the lipid A domain of bacterial LPS, forming a stable complex that blocks LPS binding to TLR4. This molecular decoy mechanism represents a novel therapeutic strategy for sepsis. LCN2 can also induce immune tolerance by upregulating human leukocyte antigen-G (HLA-G) on healthy human CD4^+^ T cells and promoting regulatory T cell (Treg) expansion (Jaworecka et al., 2021). Conversely, reduced Treg numbers may trigger autoimmunity, though the role of elevated LCN2 in autoimmune diseases requires further clarification.

Since its initial identification as NGAL, LCN2 research has evolved from phenomenological observation to mechanistic analysis, firmly establishing its central role in inflammation regulation. Early work identified LCN2 as a downstream target of NF-κB signaling, with cytokines like IL-1β upregulating its expression in inflammatory cells and parenchymal organs to form a positive feedback loop (Veeriah et al., 2016). Subsequent research has revealed highly diverse functional genealogies for LCN2.

In metabolic diseases, LCN2 exhibits a unique regulatory pattern. Studies using liver-specific knockout models demonstrate that LCN2 regulates triglyceride storage and mobilization balance by stabilizing lipid droplet surface proteins in hepatocytes, playing a dual role in fatty liver disease onset and progression (Xu et al., 2019). Concurrently, LCN2 enhances ATP-binding cassette transporter A1 (ABCA1) activity, promoting reverse cholesterol transport to the liver and accelerating high-density lipoprotein generation (Zannis et al., 2015), thereby offering a potential intervention target for atherosclerosis. Osteoblast-derived LCN2 maintains glucose homeostasis by inducing insulin secretion, improving glucose tolerance and insulin sensitivity, and suppressing feeding behavior (Mosialou et al., 2017). It crosses the blood-brain barrier to bind the melanocortin 4 receptor (MC4R) in hypothalamic neurons, activating an MC4R-dependent anorexigenic pathway (Mosialou et al., 2017). LCN2 also exacerbates insulin resistance by affecting serine phosphorylation of insulin receptor substrate-1 (IRS-1) (Xiao et al., 2017), while in obesity-related adipose tissue remodeling, it modulates preadipocyte differentiation and adipokine secretion to regulate systemic metabolic homeostasis (Yan et al., 2007).

In tumor biology, LCN2 has dual effects in hepatocellular carcinoma. It can induce a Caspase cascade in hepatoma cells by activating the mitochondrial apoptosis pathway; conversely, LCN2 in the tumor microenvironment promotes polarization of tumor-associated macrophages toward an M2 phenotype, fostering an immune microenvironment conducive to tumor growth. This bidirectional regulation complicates precise therapeutic targeting in liver cancer.

Crucially, the biological function of LCN2 exhibits a duality that depends on the disease stage and physiological context. Under homeostatic conditions, LCN2 acts as a stress-response protein that maintains systemic stability by sequestering iron and bolstering innate immunity. During acute injury, LCN2 can exert a protective, compensatory effect; for example, its acute upregulation in renal pathophysiology limits oxidative damage and facilitates cellular repair. However, when inflammation becomes chronic, sustained high levels of LCN2 become maladaptive. This functional switch analogous to its role in chronic kidney disease, where it acts as a pro-fibrotic factor and occurs in the nervous system. In chronic pain and itch, persistent LCN2 secretion transitions from a protective immune response into a pathological driver of central sensitization, exacerbating neuronal hyperexcitability and fostering a neurotoxic microenvironment. Distinguishing these homeostatic from pathological actions is therefore essential for developing time-specific therapeutic strategies that target LCN2 only when its actions become detrimental.

In neurobiology, recent studies reveal a potential role for LCN2 in sensory regulation. Clinical cohort data show a significant positive correlation between serum LCN2 levels and pruritus Visual Analogue Scale scores in psoriasis patients, suggesting LCN2 as a potential biomarker for subjective symptom intensity (Aizawa et al., 2019). In neuropathic pain models, peripheral nerve injury upregulates LCN2 expression, activating an LCN2-chemoattractant pathway that promotes spinal microglial activation and drives pain sensitization; genetic deletion or antibody neutralization of LCN2 alleviates pain-like behaviors (Jeon et al., 2013). Activated microglia secrete LCN2 via an NF-κB-dependent pathway, and LCN2 disrupts neuronal iron homeostasis through iron chelation while promoting oxidative stress product accumulation, creating a neurotoxic microenvironment that exacerbates neuronal damage (Ghosh et al., 2020). At the central level, elevated LCN2 in anterior cingulate cortex neurons enhances excitatory synaptic transmission, maintaining central sensitization in chronic pain (Song et al., 2023). These mechanisms provide preclinical evidence for LCN2 as a potential therapeutic target in neuropathic pain.

## 4 LCN2 and chronic pain

LCN2 has evolved from a classical biomarker of inflammation to a recognized gliotransmitter that orchestrates the transition from acute nociception to chronic pain states. While traditionally viewed through the lens of innate immunity, recent evidence positions LCN2 as a critical bridge connecting neuroinflammation with neuronal excitability. In the spinal dorsal horn and supraspinal centers, LCN2 acts as a molecular “gain control,” amplifying nociceptive transmission by modulating iron homeostasis, inducing oxidative stress, and reshaping synaptic plasticity. Distinguishing functionally validated receptor interactions in sensory research from those inferred from other physiological systems is critical. Direct experimental evidence from rodent models currently supports the involvement of 24p3R (NGALR) and MC4R in chronic pain and pruritus. Specifically, 24p3R-mediated NF-κB activation in spinal astrocytes drives neuropathic hypersensitivity, while keratinocyte-derived LCN2 acts directly on neuronal MC4R to trigger pruritic behaviors. In contrast, the roles of LRP2 and LRP6 in the nervous system remain largely putative. Although LRP2 is a major endocytic receptor for LCN2 in the kidney, its functional contribution to neuroimmune signaling in the spinal cord or brain has not been definitively established in pain or itch models. Similarly, while LRP6 interacts with LCN2 to modulate Wnt signaling in mesenchymal cells, this axis remains unvalidated in the context of neural sensitization. Future studies employing cell-type-specific receptor knockouts are required to confirm whether these systemic receptors play irreplaceable roles in processing noxious stimuli. This section dissects the distinct yet converging roles of LCN2 across inflammatory, neuropathic, and central pain syndromes.

### 4.1 LCN2 and neuropathic pain

**FIGURE 2:**
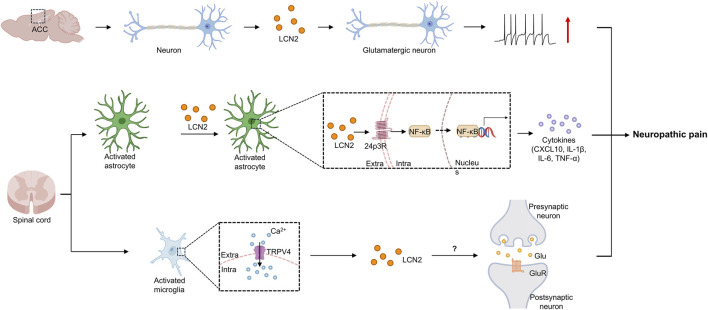
LCN2-mediated neuropathic pain and associated molecular mechanisms. Nerve injury induces the upregulation of LCN2 expression in ACC neurons. Elevated LCN2 directly acts on glutamatergic neurons, maintaining central pain sensitization by enhancing excitatory synaptic transmission and increasing firing frequency. In the spinal dorsal horn, astrocytes are activated and secrete LCN2. LCN2 binds to its specific receptor 24p3R, activating the downstream NF-κB signaling pathway. This signaling axis induces the transcription and release of pro-inflammatory cytokines (such as CXCL10, IL-1β, IL-6, and TNF-α), significantly amplifying the neuroinflammatory response. Peripheral nociceptive signals activate microglia through calcium influx mediated by TRPV4 ion channels. LCN2 released by TRPV4^+^ microglia enhances the functional and structural plasticity of excitatory spinal neurons. CXCL10, C-X-C motif chemokine ligand 10; IL-1β, Interleukin-1 betaIL-6, interleukin-6; TNF-α, tumor necrosis factor alpha; TRPV4, transient receptor potential vanilloid 4.

Neuropathic pain is driven by “central autonomy” where sensitization persists independently of peripheral inputs (Li Y. C. et al., 2025). Expressed in astrocytes, LCN2 contributes substantially to the initiation and maintenance of neuropathic pain by driving central sensitization through the regulation of neuroinflammation and neuron-glia interactions (Lu and Gao, 2023; Liu et al., 2025). Its mechanisms are multifaceted (Huang et al., 2020; Lu et al., 2021; Han et al., 2025; Lu et al., 2025). LCN2 can bind the 24p3R receptor to activate the downstream NF-κB signaling pathway, which promotes the release of inflammatory factors and amplifies neuroinflammatory responses (Jiang et al., 2020; Kong et al., 2021; Ma et al., 2025) (Figure 2). Alternatively, LCN2 can bind iron and perturb local iron metabolism; excessive iron accumulation may induce oxidative stress, causing neuronal and glial damage that exacerbates pain. Furthermore, LCN2 may regulate the transmission and integration of pain signals by influencing neuronal synaptic plasticity and altering the strength of interneuronal connections (Doliwa et al., 2025). LCN2 is pivotal in establishing this autonomous state by orchestrating a vicious cycle of neuron-glia interaction.

Activation of the TRPV4 ion channel in spinal microglia is a key event in the development of neuropathic pain (Hu et al., 2023). The TRPV4-dependent neuro-immune axis plays a crucial role in its onset and progression, as both global and microglia-specific *Trpv4* knockout and pharmacological inhibition of TRPV4 significantly alleviate neuropathic pain behaviors in mice. Mechanistic studies reveal that TRPV4 activation promotes LCN2 secretion from microglia. Secreted LCN2 then exacerbates nociceptive transmission by enhancing the excitability and synaptic plasticity of spinal neurons, thereby mediating neuropathic pain (Hu et al., 2023). These findings establish LCN2 as a molecular pain mediator and identify a key molecular switch that converts microglia from an immune-surveillance state to a pain-modulatory state.

Crucially, recent high-resolution imaging has clarified the structural basis of this plasticity. Doliwa et al. demonstrated that astrocyte-secreted LCN2 is not merely a chemical modulator but a morphological architect. It directly increases the density of mature dendritic spines in excitatory neurons, providing the physical substrate for the “maladaptive memory” of pain.

In the anterior cingulate cortex (ACC), a key region for the affective processing of pain, altered LCN2 levels directly influence pain chronicity (Song et al., 2023). Elevating LCN2 in the mouse ACC increases the excitability of ACC glutamatergic neurons and heightens pain sensitivity, whereas reducing LCN2 decreases neuronal excitability and alleviates chronic pain. In a model of remifentanil-induced postoperative hyperalgesia, ACC LCN2 levels are also significantly elevated and correlate closely with hyperalgesic behavior; inhibiting LCN2 signaling markedly reduces hyperalgesia (Shen et al., 2025).

Beyond spatial regulation, recent evidence suggests that LCN2 also exerts a “temporal dimension” of influence on neuropathic pain. Cancer-induced tactile allodynia involves significant nerve damage and compression and often exhibits circadian rhythm fluctuations. Yasukochi et al. found that spinal LCN2 is a molecular driver of this rhythmicity. In a fibrosarcoma model, the expression of LCN2 in spinal microglia is not constant but fluctuates periodically and is regulated by the circadian clock component REV-ERB. Under pathological conditions (e.g., high levels of IL-6), the inhibitory effect of REV-ERB on LCN2 is disrupted, leading to time-dependent peaks in LCN2 expression and correlating with hyperalgesia (Yasukochi et al., 2025). This reveals a “clock-immune-pain” axis, indicating that the maintenance of neuropathic pain is dynamic and closely linked to the host’s circadian biology.

The regulation of microglial function by LCN2 exhibits conserved features across pathologies. LCN2 plays a pivotal role in microglial dysfunction after experimental intracerebral hemorrhage, where its overexpression or silencing substantially alters microglial activity (Fei et al., 2024). This indicates that LCN2 is critically involved in neuropathic pain progression, particularly in spinal microglial activation and the enhancement of neuronal excitability. Future studies should therefore explore LCN2 as a potential therapeutic target for neuropathic pain and clarify how it mediates biological effects by regulating neuroplasticity.

Existing evidence supports a multi-level regulatory framework for LCN2-mediated neuropathic pain: peripheral noxious stimuli activate the TRPV4 channel in spinal microglia, inducing LCN2 secretion. LCN2 then enhances neuronal excitability at the spinal level and, via ascending projections, influences cortical pain-processing centers, ultimately forming a spinal-cortical pain-amplification loop. The completeness of this network, however, requires further validation. Key unresolved questions include the specificity of LCN2’s role across pain subtypes, its molecular interactions with classical pain mediators, and the potential consequences of long-term LCN2 inhibition on overall nervous system function.

Recent integrative proteomics analyses further substantiate the pivotal role of LCN2 in the neuroinflammatory landscape of neuropathic pain (Majumdar et al., 2026). These studies demonstrate that neuropathic pain development entails a large-scale reorganization of the spinal and hippocampal proteomes, characterized by significant dysregulation of proteins involved in immune response, synaptic plasticity, and metabolic regulation. Within these proteomic networks, LCN2 acts as a central node, coordinating with other differentially expressed proteins to drive the progression from acute injury to chronic neuroinflammation. This system-level perspective reveals that LCN2-mediated neuroimmune interactions are not isolated events but integral components of a broader molecular signature of chronic pain. Consequently, targeting such central regulators appears essential for achieving effective analgesia.

A defining mechanistic feature of LCN2-mediated neuropathic pain is the convergence of microglial signaling, transcriptional regulation, and metabolic dysregulation on neuronal function. The cascade is initiated by the activation of TRPV4 channels in spinal microglia, which serves as a primary stimulus for LCN2 secretion. Upon release, LCN2 disrupts neuronal stability through two principal pathways. First, it activates the NF-κB signaling axis in both glial cells and neurons, thereby promoting a pro-inflammatory microenvironment that lowers the firing threshold of nociceptive fibers. Second, functioning as a high-affinity iron-binding protein, LCN2 induces significant iron dysregulation and accumulation within the spinal dorsal horn. The resulting intracellular iron overload fosters oxidative stress and may trigger ferroptosis, which, together with NF-κB-driven cytokine release, establishes a state of persistent neuronal hyperexcitability. This synergistic framework establishes the TRPV4-LCN2-Iron axis as a core molecular engine driving the transition from neuroinflammation to chronic pain.

### 4.2 LCN2 and inflammatory pain

Inflammatory pain typically arises from tissue injury or inflammatory responses, and its development is closely linked to the activation of local immune cells and the release of inflammatory mediators (Ji et al., 2016; Kong et al., 2021; Lu and Gao, 2023). In this context, LCN2 operates as a key metabolic modulator. Studies indicate that LCN2 is secreted by activated astrocytes and microglia during neuroinflammation and participates in regulating cell survival and inflammatory signaling (Jin et al., 2014). In the experimental autoimmune encephalomyelitis (EAE) model, LCN2-deficient mice showed more severe disease progression, suggesting that LCN2 may provide a protective effect by restraining excessive pro-inflammatory responses (Gasterich et al., 2022). Conversely, in models of spinal cord injury or cerebral ischemia, LCN2 expression is markedly increased, where it promotes immune cell infiltration, exacerbates neuronal loss, and aggravates subsequent inflammatory responses and neurological dysfunction (Rathore et al., 2011).

Mechanistically, this exacerbation is driven by metabolic vulnerability. LCN2 disrupts cellular iron homeostasis, a process central to its pathological function. Ferroptosis is a regulated form of cell death driven by iron-dependent lipid peroxidation (Du et al., 2021). Under inflammatory conditions, LCN2 accumulation dysregulates the intracellular labile iron pool (LIP), sensitizing neurons to oxidative stress and ferroptosis—an iron-dependent form of cell death. Xiao et al. demonstrated that LCN2 deficiency mitigates this cascade, reducing NLRP3 inflammasome activation and oxidative damage (Xiao et al., 2017; An et al., 2023; Jiang et al., 2024; Zhao et al., 2024). In a dextran sulfate sodium (DSS)-induced colitis model, LCN2 limits iron toxicity by promoting hypoferremia (Li et al., 2023). Its downstream effects on the NF-κB/STAT3 axis in the nervous system ultimately fuel neuronal ferroptosis and lipid peroxidation (Deng et al., 2023; Jiang et al., 2025).

Astrocyte-derived LCN2 acts as a secondary relay in this process. Research by Activated by peripheral cytokines, spinal astrocytes secrete LCN2, which enhances neurotoxicity and suppresses neurotrophic support (Shao et al., 2022; Jung et al., 2023). Mechanistically, LCN2 exacerbates pain by promoting pro-inflammatory cytokine release and suppressing neurotrophic factor expression. Astrocyte-derived LCN2 enhances neurotoxicity in chronic pain through NF-κB pathway activation, implying that inhibiting LCN2 secretion could alleviate neuroinflammation (Mongre et al., 2016; Tang et al., 2018; Coorens et al., 2019). This creates a metabolic environment where neurons are primed for hyperexcitability. Furthermore, the upregulation of LCN2 extends to supraspinal centers like the ACC, where it directly drives neuronal sensitization (Song et al., 2023). Thus, in inflammatory pain, LCN2 functions as a metabolic stressor that converts immune signals into neuronal vulnerability (Petropoulou et al., 2020).

Beyond classical inflammation, recent studies have unveiled the sex-specific and metabolic dimensions of LCN2 signaling. First, sexual dimorphism is a critical but often overlooked factor. Pepino et al. reported that in formalin-induced inflammatory pain, LCN2 expression displays a distinct male preponderance. Transcriptomic analysis revealed that the recruitment of LCN2-expressing neutrophils to the spinal meninges is a key driver of pain in males, whereas females rely on alternative mechanisms potentially linked to the estrus cycle (Pepino et al., 2023; Xie et al., 2025). This suggests that LCN2-targeted therapies might need to be sex-stratified. Second, LCN2 acts as a bridge between viral infection and metabolic pain. In a model of Enterovirus-A71 (EV-A71) infection, astrocyte-derived LCN2 was found to trigger lactate accumulation in muscle tissue. This metabolic shift, driven by the HMGB1-LCN2 axis, directly sensitizes peripheral nociceptors, identifying a novel “brain-muscle” metabolic loop that underlies viral myalgia (You et al., 2025).

Both inflammatory and neuropathic pain involve LCN2-mediated neuroimmune signaling, yet their etiologies and regulatory mechanisms differ significantly. Inflammatory pain arises primarily from a robust peripheral immune response, where tissue injury or infection recruits neutrophils and macrophages that secrete LCN2 at the site of inflammation. Within this local inflammatory milieu, LCN2 sensitizes nociceptors, transiently amplifying acute peripheral signals. Peripheral inflammatory factors such as IL-6 subsequently activate spinal astrocytes via axonal transport. Neuropathic pain, in contrast, centers on autonomous central sensitization. Nerve injury directly induces astrocyte activation and sustained high-level LCN2 expression. After nerve injury, LCN2 upregulation depends less on systemic immune infiltration and more on the sustained activation of spinal microglia and astrocytes, positioning LCN2 as a chronic neuromodulator that links glial activation to synaptic plasticity and oxidative stress. Notably, early microglial activation in neuropathic pain further promotes LCN2 expression, a phenomenon not observed in inflammatory pain. Thus, in inflammatory pain, LCN2 acts as a dynamic response to external injury, while in neuropathic pain, it sustains a maladaptive, self-reinforcing hyperexcitable network.

In inflammatory pain, LCN2 signaling is largely peripheral drive-dependent and remains potentially reversible once the primary tissue injury is resolved. Here, LCN2 functions as a secondary relay for peripheral cytokines that reach the central nervous system. In contrast, neuropathic pain is defined by centrally autonomous sensitization, where LCN2 becomes a self-sustaining driver of pathology. In this autonomous state, persistent LCN2 secretion from activated glia maintains a “maladaptive memory” of pain by increasing mature dendritic spine density and inducing neuronal hyperexcitability. This explains why neuropathic pain exhibits greater chronicity and treatment resistance compared to pure inflammatory pain.

### 4.3 LCN2 in morphine tolerance and hyperalgesia

Morphine is the most widely used opioid analgesic in clinical practice, demonstrating significant efficacy for both acute and chronic pain. However, long-term administration inevitably leads to tolerance and opioid-induced hyperalgesia (OIH), which substantially diminish its analgesic efficacy and limit its clinical utility. Neuroinflammation and central sensitization are now recognized as pivotal mechanisms in this process. LCN2, an acute-phase protein, plays a significant regulatory role in inflammatory responses, glial cell activation, and synaptic plasticity. Notably, LCN2 contributes not only to enhanced nociceptive transmission after nerve injury but also appears central to the development of tolerance and hyperalgesia from prolonged morphine use.

**FIGURE 3:**
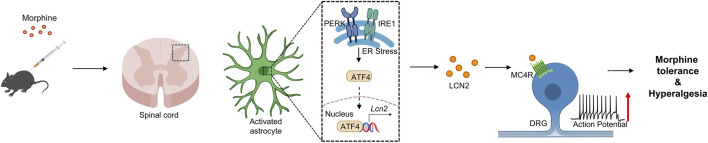
LCN2-mediated morphine tolerance and hyperalgesia associated molecular mechanisms. Chronic or repeated use of morphine induces endoplasmic reticulum stress (ER stress) in spinal dorsal horn astrocytes. This occurs through the activation of the PERK and IRE1 signaling pathways, leading to the nuclear translocation of the downstream transcription factor ATF4 and promoting the expression of *Lcn2*. The upregulated LCN2 further binds to the melanocortin 4 receptor (MC4R) and mediates downstream signal transduction. This LCN2-MC4R signaling axis enhances the excitability of spinal neurons, ultimately leading to morphine tolerance and hyperalgesia. PERK, PKR-like endoplasmic reticulum kinase; IRE1, inositol-requiring enzyme 1.

Recent work has further elucidated the critical role of astrocyte-derived LCN2 in morphine tolerance and hyperalgesia (Figure 3). Wang et al. demonstrated that repeated intrathecal morphine injections induce endoplasmic reticulum (ER) stress in spinal dorsal horn astrocytes. This ER stress, mediated via the PERK and IRE1 pathways, activates the downstream transcription factor ATF4, leading to a significant upregulation of LCN2 expression (Wang et al., 2024). The elevated LCN2 promotes NLRP3 inflammasome activation and enhances spinal neuron hyperexcitability, while also directly modulating neuronal firing via the MC4R signaling pathway, ultimately contributing to hyperalgesia.

Functional experiments showed that inhibiting the PERK/IRE1 pathways or using small interfering RNA to suppress LCN2 effectively alleviated morphine tolerance and hyperalgesia without affecting morphine’s acute antinociceptive effects. This finding indicates that LCN2-mediated neuron-glia interactions are a core mechanism in the initiation and progression of morphine tolerance (Wang et al., 2024). The study underscores the critical position of the astrocytic ER stress-LCN2 axis in morphine-related pathological pain and suggests that modulating LCN2 signaling could preserve analgesic efficacy while circumventing tolerance. This provides a novel perspective for safer clinical opioid use.

The clinical management of chronic pain is frequently hindered by the paradoxical development of OIH and analgesic tolerance, which necessitate higher doses and increase the risk of respiratory depression and opioid use disorder. Current evidence positioning LCN2 as a key mediator of these processes offers a promising clinical strategy. Unlike traditional adjuvants that may interfere with opioid receptor signaling, targeting the astrocytic ER stress-LCN2 axis specifically addresses the neuroinflammatory and metabolic underpinnings of tolerance without compromising the acute antinociceptive effects of morphine. From a translational perspective, the observation that serum LCN2 concentrations are significantly elevated in opioid-tolerant patients suggests its potential as a clinical biomarker to monitor the onset of tolerance. Furthermore, pharmacological inhibition of ER stress sensors or LCN2 signaling has been shown to potentiate morphine analgesia and attenuate hypersensitivity without affecting its acute antinociceptive benefits. These findings indicate modulating the astrocyte ER stress-LCN2 axis could preserve the clinical utility of opioids while circumventing their maladaptive neuroplastic consequences.

### 4.4 LCN2 and stroke-associated pain

**FIGURE 4:**
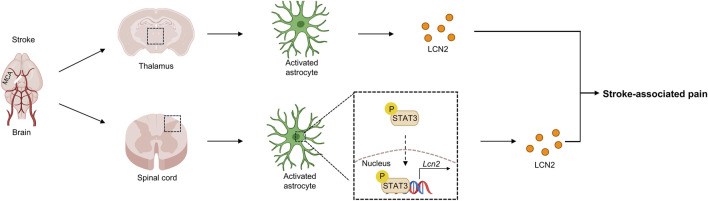
LCN2-mediated stroke-associated pain and associated molecular mechanisms. Following ischemic stroke, activated astrocytes in the damaged thalamus directly secrete LCN2, contributing to the local inflammatory environment. The stroke also induces astrocyte activation at the spinal cord level, driving the transcription and expression of Lcn2 through phosphorylation of the STAT3 pathway (p-STAT3). The combined action of LCN2 from both the thalamus and spinal cord amplifies the transmission of pain signals, promoting the persistent maintenance of Stroke-associated pain. MCA, middle cerebral artery.

Stroke-associated pain, clinically termed Central Post-Stroke Pain (CPSP), is a debilitating neuropathic pain syndrome resulting from cerebrovascular accidents in the somatosensory pathway. It is characterized by persistent intractable pain, allodynia, and hyperalgesia, leading to a poor prognosis (Figure 4). A growing body of research indicates that the thalamus plays a central role in the initiation and maintenance of central pain, with neuroinflammation and abnormal glial cell activation considered key pathological mechanisms. LCN2, an inflammation-associated molecule, is rapidly upregulated following central nervous system injury. It participates in the amplification and persistence of pain signals by modulating the activation of microglia and astrocytes, promoting the release of inflammatory factors, and mediating abnormal remodeling of neural networks. In models of thalamic pain, evidence suggests that high expression of LCN2 is closely associated with pain sensitization (Li Y.-R. et al., 2025). Its effects may be realized by driving neuroinflammatory responses and disrupting the excitatory-inhibitory balance within the thalamus.

Studies have shown that upregulated expression of LCN2 in the spinal dorsal horn is a key factor in the occurrence and development of CPSP (Nakamoto and Tokuyama, 2024). In CPSP model mice, astrocytes are activated in the spinal cord, and LCN2 expression is upregulated in the dorsal horn. GFAP-positive cells in the mouse spinal cord co-express STAT3. These responses can be blocked by AG490 (an inhibitor of JAK2 and downstream STAT3 activation) or by anti-LCN2 antibodies. By promoting glial cell activation and inflammatory factor release, LCN2 drives central sensitization and abnormal amplification of pain signals, providing new molecular evidence for the maintenance of central pain.

Further research has validated the critical role of LCN2 at the thalamic level. Li et al. found that in a mouse model of thalamic pain, the level of LCN2 secreted by astrocytes was significantly elevated (Li Y.-R. et al., 2025). This change was closely correlated with central inflammatory responses and pain-related behavioral manifestations. The excessively secreted LCN2 promoted the occurrence and maintenance of thalamic pain by amplifying inflammatory signaling between glial cells and inducing synaptic plasticity abnormalities. Astrocyte-derived LCN2 is not merely an inflammatory marker but directly participates in the pathological process of pain sensitization. Building on this, the study further revealed that the natural product obacunone could enhance LCN2-mediated phagocytic function in astrocytes, thereby attenuating abnormal inflammatory signaling and neural circuit imbalance, leading to significant alleviation of thalamic pain. These results suggest that astrocyte-secreted LCN2 exerts a pro-nociceptive effect in thalamic pain, and modulating LCN2 signaling may represent a potential therapeutic strategy.

**FIGURE 5:**
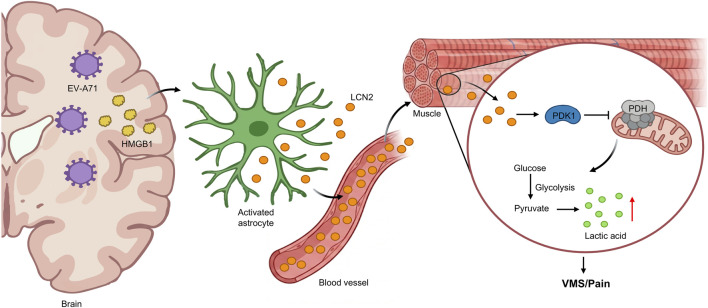
LCN2-mediated virus-associated muscle soreness and associated molecular mechanisms. EV-A71 infection of the brain induces astrocytes to release LCN2 into the bloodstream via HMGB1 signaling. Circulating LCN2 reaches muscle tissue, upregulating PDK1 activity and inhibiting pyruvate dehydrogenase (PDH). This metabolic blockade leads to the conversion of glucose glycolysis products into large amounts of lactate, directly sensitizing peripheral nociceptors and inducing virus-associated muscle soreness (VMS/Pain). HMGB1, high mobility group box 1; PDK1, pyruvate dehydrogenase kinase 1.

In addition, LCN2 also acts as a bridge between viral infection and metabolic pain. A recent study in Science Advances expanded the pathogenic scope of LCN2 to viral-induced myalgia. In an EV-A71 infection model, astrocyte-derived LCN2 was found to trigger lactate accumulation in muscle tissue (Figure 5). This metabolic shift directly sensitized nociceptors, identifying a novel “Virus-Astrocyte-LCN2-Lactate” axis (You et al., 2025). This finding reinforces the concept that LCN2 serves as a metabolic disruptor, whether via iron handling or lactate production—to lower the nociceptive threshold.

In summary, LCN2 plays a pivotal role in both the initiation and progression of pain. Its mechanisms involve neuro-glia interactions, regulation of neuroinflammatory responses, maintenance of iron metabolism homeostasis, and modulation of neuronal excitability. Through interactions with glial cells and neurons, LCN2 not only promotes the sustained release of inflammatory factors and alterations in synaptic plasticity but may also exacerbate neuronal damage and pain sensitization by disrupting iron homeostasis and enhancing oxidative stress. Given its important position in multiple pathological processes, intervention strategies targeting LCN2 or its downstream signaling pathways (e.g., NF-κB, STAT3) hold promise for breaking the vicious cycle between inflammatory responses and abnormal neural plasticity. This provides new insights and potential therapeutic targets for the prevention and treatment of inflammatory pain and neuropathic pain. Future studies should further evaluate the specific role of LCN2 in different pain models and explore the feasibility and safety of its clinical translation.

## 5 LCN2 and chronic pruritus

Pruritus is categorized as either acute or chronic. Acute pruritus, which typically arises over days to weeks, is frequently triggered by allergic reactions, infections, or physical stimuli. Although its symptoms can be severe, they usually resolve quickly and serve a biological warning function. Chronic pruritus, defined as lasting more than 6 weeks, has complex etiologies that can include dermatological diseases, systemic disorders, neuropathies, or psychological factors. Its recurrent and protracted symptoms substantially impair patients’ quality of life. LCN2 promotes the development and maintenance of chronic pruritus by enhancing neuroinflammation and glial cell activation, thereby amplifying the transmission of pruritic signals.

### 5.1 LCN2 and itch in allergic contact dermatitis (ACD)

Allergic contact dermatitis (ACD) is a delayed-type hypersensitivity skin disease triggered by contact allergens, characterized clinically by local erythema, vesicles, exudation, and intense pruritus. Its pathophysiology comprises two distinct phases: sensitization and elicitation. During sensitization, haptens conjugate with skin proteins to form complete antigens, which are captured by Langerhans cells and presented to T cells. Upon re-exposure to the antigen in the elicitation phase, sensitized T cells become rapidly activated and release a large array of inflammatory cytokines, driving the local inflammatory response and tissue damage. In ACD mouse models, while the expression level of the gastrin-releasing peptide receptor (GRPR) remains unchanged, the sensitivity of GRPR^+^ dorsal horn neurons to gastrin-releasing peptide (GRP) is significantly enhanced (Koga et al., 2019). Mechanistic investigations have revealed that this heightened sensitivity stems from astrocyte activation and their subsequent secretion of LCN2. Inhibiting astrocyte activation or specifically knocking out the *Lcn2* gene in astrocytes significantly attenuates the GRP-induced increase in neuronal excitability while concurrently improving scratching behavior and skin lesions. Notably, even in normal mice, exogenous LCN2 can potentiate GRP-induced neuronal excitability. Collectively, these findings indicate that under chronic itch conditions, LCN2 modulates neuronal functional state via a non-cell-autonomous mechanism, independent of alterations in receptor expression levels.

**FIGURE 6:**
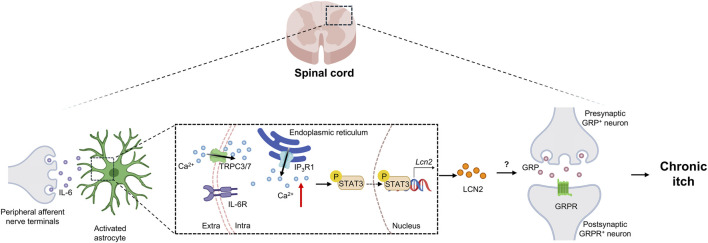
The central molecular mechanism of LCN2-mediated chronic pruritus. In a murine model of chronic dermatitis, IL-6 released from DRG neurons binds to IL-6 receptors on spinal cord astrocytes, thereby activating an IP_3_R1/TRPC-dependent Ca^2+^ signaling pathway. This sustained astrocytic Ca^2+^ signaling drives persistent STAT3 activation and a marked upregulation of LCN2 expression. The elevated LCN2 then enhances the excitability of spinal GRPR^+^ neurons, which promotes the development and maintenance of chronic itch. DRG: dorsal root ganglion; IL-6, interleukin-6; IL-6R, interleukin-6 receptor; TRPC3/7, transient receptor potential canonical 3/7; IP3R1, inositol 1,4,5-trisphosphate receptor type 1; GRP, gastrin-releasing peptide; GRPR, gastrin-releasing peptide receptor.

From a signal transduction perspective, the IL-6/STAT3/LCN2 axis plays a crucial role in chronic itch. In ACD models, Ca^2+^ imaging studies using primary astrocytes and spinal cord slices from IP_3_R1 knockout mice demonstrated that IL-6, by activating the IP_3_R1/TRPC-dependent Ca^2+^ signaling pathway in astrocytes, induces sustained STAT3 activation and upregulates LCN2 expression, thereby enhancing the excitability of GRPR^+^ neurons and promoting chronic itch (Shiratori-Hayashi et al., 2021) (Figure 6). The compound 3-oxa-PD1 exhibits significant anti-pruritic efficacy across diverse mouse models (Furutani et al., 2023), including histamine-induced acute itch, chloroquine-induced non-histaminergic itch, morphine-induced central itch, cutaneous T-cell lymphoma (CTCL)-induced chronic itch, and 2,4-dinitrofluorobenzene (DNFB)-induced ACD and imiquimod (IMQ)-induced psoriasis models. Under pruritic conditions, activated astrocytes release LCN2, which drives neuroinflammation and synaptic plasticity to exacerbate itch sensation; 3-oxa-PD1 restores neural circuit homeostasis by inhibiting LCN2 secretion.

In a chronic itch model induced by topical application of diphenylcyclopropenone (DCP) to establish ACD, baicalein dose-dependently inhibited the chronic itch response and significantly alleviated skin lesions at the application site (Du et al., 2025). Intrathecal injection of the STAT3 inhibitor AG490 or LCN2 siRNA significantly reduced scratching behavior and astrocyte activation in ACD mice. Furthermore, baicalein markedly attenuated the expression of p-STAT3 and LCN2 in the spinal cords of ACD mice and in lipopolysaccharide-stimulated primary spinal astrocytes (Figure 6). These results indicate that baicalein alleviates chronic itch by modulating the spinal astrocyte STAT3-LCN2 pathway.

In summary, current research has delineated a coherent pathological cascade in ACD itch: “peripheral immune activation - IL-6 release - astrocyte activation - LCN2 secretion - neuronal sensitization.” These findings elucidate the central mechanisms underlying ACD itch and provide a theoretical foundation for developing novel anti-pruritic therapies targeting LCN2.

### 5.2 LCN2 and pruritus in xerosis

Xerosis is a common dermatological condition characterized by impaired skin barrier function, leads to transepidermal water loss, inflammatory responses, and pruritus. In an acetone-ether-water (AEW)-induced xerosis model, paeonol significantly inhibits CXCR3 expression in spinal cord neurons and downregulates AEW-induced astrocyte activation markers, including *Tlr4*, *Lcn2*, and *Hspb1* (Shao et al., 2019). The inhibitory effect of paeonol on AEW-induced scratching behavior and spinal astrocyte activation is abolished by CXCR3 antagonism or genetic knockout (Roudkenar et al., 2007; Lee E. K. et al., 2011). Thus, targeting LCN2 or its signaling pathway represents a potential therapeutic strategy for xerosis-associated pruritus.

LCN2 is critically involved in the pathophysiology of chronic pruritus and is a key target for its intervention. It regulates chronic pruritus signal transmission in astrocytes via a STAT3-dependent mechanism, with its expression level correlating closely with pruritus severity (Yang et al., 2022). LCN2 is secreted not only by glial cells but also by keratinocytes and neutrophils, where it participates in modulating pruritus signaling. Its expression is significantly elevated in patients with chronic pruritus, particularly in those with chronic kidney disease, where increased serum and tissue LCN2 levels positively correlate with pruritus severity (Bazid et al., 2023). The role of LCN2 in glial cells may involve the regulation of calcium signaling and TRPC channel activity, thereby influencing sensory neuron sensitivity.

**FIGURE 7:**
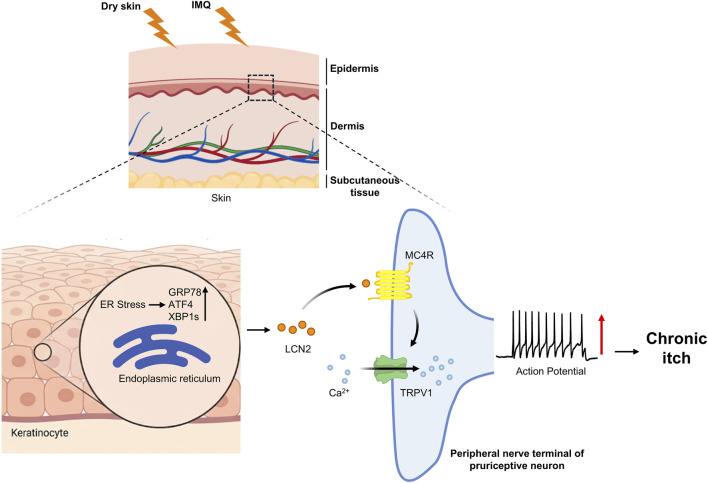
Peripheral molecular mechanisms of LCN2-mediated chronic pruritus. Under endoplasmic reticulum stress (upregulation of GRP78, ATF4, XBP1s.), keratinocytes secrete LCN2. The secreted LCN2 acts on MC4R receptors in DRG neurons via blood circulation or local diffusion, directly inducing intense scratching behavior through TRPV1 channels. GRP78, glucose-regulated protein 78; ATF4, activating transcription factor 4; XBP1s, spliced X-box binding protein 1.

Beyond spinal mechanisms, recent breakthroughs have decoded the precise signaling at the peripheral skin-nerve interface. Ge et al. identified a novel “Keratinocyte-to-Neuron” axis driving chronic itch (Ge et al., 2026). They found that Endoplasmic Reticulum (ER) stress in keratinocytes acts as the primary trigger for LCN2 secretion. Crucially, this keratinocyte-derived LCN2 functions as a direct pruritogen: it binds to the Melanocortin 4 Receptor (MC4R) on Dorsal Root Ganglion (DRG) neurons, which subsequently sensitizes TRPV1 channels to induce robust scratching behaviors (Figure 7). This finding is pivotal because it establishes LCN2 as a direct activator of peripheral nociceptors, distinct from its role in spinal gliotransmission.

### 5.3 LCN2 and pruritus in atopic dermatitis (AD)

In contrast to the central sensitization mechanism in ACD, the role of LCN2 in AD is predominantly linked to peripheral barrier dysfunction and metabolic dysregulation. AD is a common, chronic, relapsing inflammatory skin disease characterized by intense pruritus, skin barrier dysfunction, and immune dysregulation. Pruritus represents the most prominent and distressing symptom for patients, often occurring as persistent or paroxysmal episodes that severely impair sleep and quality of life. Arginase 1 (ARG1), a key enzyme in arginine metabolism, also regulates LCN2 expression through its metabolites, thereby influencing keratinocyte differentiation and skin barrier integrity in ways essential for maintaining skin homeostasis. In AD models, ARG1 expression is significantly downregulated, indicating its dysfunction may be closely linked to the skin barrier defects observed in AD (Szondi et al., 2025). ARG1 catalyzes the conversion of arginine to ornithine, which is then metabolized to produce polyamines and urea. These metabolites can modulate LCN2 expression levels, revealing an intrinsic connection between the arginine metabolic pathway and LCN2 regulation (Szondi et al., 2025). LCN2 expression increases during keratinocyte differentiation, where it contributes to skin barrier function, and its level may serve as a potential biomarker for assessing barrier status (Su et al., 2021). Furthermore, as an antimicrobial peptide, LCN2 expressed during keratinocyte differentiation is vital for skin immune defense and may be regulated via the ARG1 metabolic pathway. In AD models, LCN2 expression is downregulated but can be restored by supplementing ARG1 metabolites (Haneda et al., 2016). This compensatory mechanism suggests that deficient metabolite production due to ARG1 dysfunction significantly contributes to reduced LCN2 expression in AD. Restoring ARG1 pathway activity or directly supplementing its metabolites can regulate the ARG1-LCN2 axis, which plays an important role in maintaining skin barrier function and immune defense under various pathological conditions.

In summary, the core mechanism of AD pruritus involves skin barrier dysfunction mediated by the ARG1-LCN2 metabolic axis. Unlike in ACD, where LCN2 primarily regulates neuronal sensitization through central astrocyte activation, LCN2 in AD acts predominantly at the peripheral skin level by affecting keratinocyte differentiation and barrier integrity. This finding expands the understanding of LCN2’s functional diversity and indicates that treatments for different skin diseases should account for the tissue specificity and functional differences of LCN2, providing a theoretical basis for precise and individualized therapy.

### 5.4 LCN2 and spontaneous dermatitis-associated pruritus

Spontaneous dermatitis comprises a group of chronic inflammatory skin diseases that arise without clear external triggers. These conditions are defined by persistent cutaneous inflammation, a compromised epidermal barrier, and intractable pruritus. Using a spontaneous dermatitis model (KCASP1Tg), Iida et al. explored the link between chronic skin inflammation and anxiety-like symptoms (Iida et al., 2023). Their study found that KCASP1Tg mice displayed significant anxiety-like behaviors alongside markedly increased *Lcn2* mRNA levels in the brain, which correlated with stimulation by the inflammatory cytokine IL-1β. Plasma LCN2 levels were also significantly elevated in these mice compared to controls. While JAK inhibitor treatment reduced plasma LCN2, it did not alleviate the anxiety-like behaviors. These results imply a close association between LCN2 and anxiety symptoms and further suggest that the anxiety- and depression-like symptoms driven by chronic skin inflammation may be partially irreversible. This irreversibility could originate from structural or functional remodeling within the central nervous system (CNS) during prolonged inflammation. Such established neuroplastic changes may persist even after peripheral inflammation is controlled. Chronic skin inflammation stimulates LCN2 production, which then acts on the CNS to potentially induce anxiety-like symptoms. The chronic skin inflammation–LCN2–anxiety axis is therefore crucial for understanding neuropsychiatric homeostasis, particularly the potential for neurological alterations under sustained inflammatory conditions. This work highlights the importance of early and aggressive control of skin inflammation to prevent anxiety onset.

Research on spontaneous dermatitis has uncovered a new role for LCN2 in chronic skin disease—that of a key peripheral-to-central signaling mediator connecting localized skin inflammation to CNS dysfunction. These findings underscore the clinical imperative for the early and proactive management of skin inflammation. They indicate that treating chronic skin diseases should involve looking beyond cutaneous symptoms to prioritize preventing and intervening early in neuropsychiatric complications. This offers a fresh theoretical basis and a systemic therapeutic perspective for managing chronic dermatological conditions.

### 5.5 LCN2 and pruritus in psoriasis

Psoriasis is a chronic inflammatory skin disease clinically characterized by erythema, scales, and pruritus. Pruritus is among the most frequent and distressing symptoms for patients, substantially reducing quality of life. Its pathophysiology involves immune dysregulation, aberrant keratinocyte proliferation, and the abundant release of inflammatory cytokines.

LCN2 plays a critical role in psoriasis initiation and progression by activating keratinocytes and modulating inflammatory responses. Serum and skin tissue levels of LCN2 are markedly elevated in patients and correlate closely with disease activity, underscoring its importance in disease progression (Aizawa et al., 2019). Through its receptor 24p3R, LCN2 activates keratinocytes and promotes the expression of inflammatory cytokines, including IL-1β, IL-23, CXCL1, and CXCL10. It also exacerbates psoriatic inflammation by activating the cholesterol synthesis pathway, which induces SREBP2 maturation and NLRC4 inflammasome activation (Ma et al., 2022).

Mechanistic studies show that LCN2 regulates keratinocyte activation via the SREBP2-NLRC4 axis and promotes inflammatory cell infiltration and proliferation. LCN2 interacts with neutrophils, T cells, and other inflammatory cells, facilitating their recruitment through pathways such as IL-23/IL-17, p38 MAPK, and ERK1/2, thereby amplifying inflammation (Hau et al., 2016). Moreover, LCN2 may drive abnormal keratinocyte proliferation and differentiation by inhibiting the synthesis of keratins, involucrin, and related structural proteins, contributing to epidermal hyperplasia and scale formation (Liu et al., 2016; Ma et al., 2022; Ren and Xia, 2022).

Therapeutically, inhibiting LCN2 or its receptor 24p3R significantly alleviates psoriasiform skin inflammation, supporting LCN2 and its associated pathways as potential therapeutic targets (Jaworecka et al., 2021). Blocking the LCN2-24p3R axis concurrently suppresses aberrant keratinocyte activation, inflammatory cell recruitment, and inflammasome activation, playing a vital role in maintaining skin immune homeostasis across pathological conditions.

LCN2 contributes to psoriasis pathogenesis through a multi-level regulatory network encompassing receptor activation, inflammasome triggering, inflammatory cell recruitment, and abnormal keratinocyte proliferation. By bridging innate and adaptive immunity, regulating keratinocyte function, and driving chronic inflammation, LCN2 represents a key target for elucidating psoriasis pathophysiology and developing new therapies.

In summary, at the central level, both AD and xerosis activate the STAT3 signaling pathway in spinal astrocytes via peripheral inflammatory cytokines such as IL-6, driving a substantial release of LCN2. This LCN2 release then enhances the excitability of GRPR^+^ neurons in a non-cell-autonomous manner. Conversely, at the peripheral level, the induction pathways of LCN2 exhibit significant disease specificity. In ACD and psoriasis, LCN2 primarily mediates a neuroimmune circuit between keratinocytes and neutrophils. In atopic dermatitis, it mediates metabolic dyshomeostasis regulated by ARG1. Furthermore, recent studies have revealed keratinocyte-neuron axis that ER stress-induced LCN2 directly activates MC4R on DRG neurons to sensitize TRPV1 (Ge et al., 2026). These findings highlight the clinical potential of LCN2 as a therapeutic target. Inhibiting LCN2 expression or blocking its pathway effectively alleviates chronic pruritus symptoms. For example, narrow-band ultraviolet B phototherapy significantly reduces LCN2 levels in the serum and tissues of chronic kidney disease patients while improving pruritus severity (Kee et al., 2023). This underscores the regulatory importance of LCN2 in chronic pruritus treatment, and its targeted intervention may offer new therapeutic options for affected patients.

## 6 Future perspectives

While recent years have seen significant progress in understanding the role of LCN2 in pain and itch, critical questions remain. For pain, the precise mechanisms of LCN2 action across different neuropathic pain types are not fully elucidated, and its interactions with other inflammatory mediators and neurotransmitters require further study. Moreover, the clinical potential of LCN2 as a therapeutic target must be validated through more rigorous clinical trials (Ören et al., 2016; Bazid et al., 2023). In itch, the mechanisms by which LCN2 contributes to various pruritic conditions are still unclear, including its signaling within neuron-glia interactions, its synergy with pruritogenic receptors like CXCR3, and its role in central sensitization. Developing specific LCN2 inhibitors or gene therapies and systematically evaluating their efficacy and safety in preclinical models is therefore a crucial research direction.

Current literature suggests LCN2 plays a dual role in the initiation and progression of both pain and itch, primarily through glial cell activation, regulation of chemokines, and receptor-mediated signaling. Interventions such as neutralizing antibodies, siRNA, and gene-editing technologies targeting LCN2 have shown promise in preclinical studies (Guo et al., 2016), though their long-term safety and translational potential need further validation. In summary, research advances have clarified the pivotal role of LCN2 in disease pathogenesis and provided new insights and potential therapeutic targets for related conditions. As mechanistic understanding deepens and intervention strategies are optimized, the clinical prospects for targeting LCN2 are likely to expand.
